# Swimming exercise reverses transcriptomic changes in aging mouse lens

**DOI:** 10.1186/s12920-024-01839-1

**Published:** 2024-03-04

**Authors:** Lin Ye, Jiayue Yuan, Shijie Zhu, Shunmei Ji, Jinhui Dai

**Affiliations:** 1 Department of Ophthalmology, West China Hospital, Sichuan University, Chengdu, China; 2 Department of Ophthalmology, Zhongshan Hospital, Fudan University, Shanghai, China; 3 School of Medicine, Tongji University, Shanghai, China

**Keywords:** Aging, Swimming exercise, Intraocular lens, *Ciart*, Transcriptomics

## Abstract

**Background:**

The benefits of physical activity for the overall well-being of elderly individuals are well-established, the precise mechanisms through which exercise improves pathological changes in the aging lens have yet to be fully understood.

**Methods:**

3-month-old C57BL/6J mice comprised young sedentary (YS) group, while aging mice (18-month-old) were divided into aging sedentary (AS) group and aging exercising (AE) group. Mice in AE groups underwent sequential stages of swimming exercise. H&E staining was employed to observe alterations in lens morphology. RNA-seq analysis was utilized to examine transcriptomic changes. Furthermore, qPCR and immunohistochemistry were employed for validation of the results.

**Results:**

AE group showed alleviation of histopathological aging changes in AS group. By GSEA analysis of the transcriptomic changes, swimming exercise significantly downregulated approximately half of the pathways that underwent alterations upon aging, where notable improvements were ‘calcium signaling pathway’, ‘neuroactive ligand receptor interaction’ and ‘cell adhesion molecules’. Furthermore, we revealed a total of 92 differentially expressed genes between the YS and AS groups, of which 10 genes were observed to be mitigated by swimming exercise. The result of qPCR was in consistent with the transcriptome data. We conducted immunohistochemical analysis on *Ciart*, which was of particular interest due to its dual association as a common aging gene and its significant responsiveness to exercise. The Protein-protein Interaction network of *Ciart* showed the involvement of the regulation of *Rorb* and *Sptbn5* during the process.

**Conclusion:**

The known benefits of exercise could extend to the aging lens and support further investigation into the specific roles of *Ciart*-related pathways in aging lens.

**Supplementary Information:**

The online version contains supplementary material available at 10.1186/s12920-024-01839-1.

## Introduction

The term ‘aging’ refers to a multitude of pathological changes that gradually accumulate over time, ultimately compromising function and resulting in a higher incidence of various diseases. Within this context, cataract stands out as one of the most prevalent ocular ailments linked to the aging process, with numerous researchers extensively documenting age-related modifications in the ocular lens. With age, a gradual succession of biochemical changes eventually result in heightened light scattering, alteration of coloration, and increased stiffness within the lens [1]. For instance, chronic oxidative damage in the aging lens can lead to a reduction in the antioxidant glutathione, resulting in the disruption of protein aggregation regulation. This, in turn, contributes to the emergence of light scattering, a phenomenon closely linked to the progression of mature nuclear cataract formation [2]. While numerous factors are involved in the aging of the lens, it remains uncertain how to mitigate the adverse effects of the aging process.

The benefits of physical activity for the human body have long been acknowledged. Earlier prospective cohort studies demonstrated that engaging in regular and moderate physical activity could potentially lower the risk of developing cataracts [3–5]. Conversely, a lower level of physical activity was linked to an increased risk of cataracts [6]. On retinal degenerative diseases, previous studies have indicated that physical activities have the potential to offer non-pharmacological advantages in the context of age-related disorders, manifesting notable reductions in oxidative stress, inflammation, and lipid accumulation [7]. However, experimental evidence demonstrating the molecular benefits of exercise in the aging lens is lacking.

To address the issue, we examined the potential effects of regular exercise on mitigating the dysregulation observed in the aging lens of mice. For this purpose, we opted for swimming as the exercise modality, given its capacity for precise adjustments in duration and intensity to suit the mouse model. Besides, we employed RNA-seq to conduct a comprehensive comparison of the lens transcriptomes among the young sedentary (YS), aging sedentary (AS), and aging exercise (AE) groups. These findings could reinforce the significance of upholding a healthy lifestyle and behaviors, and hold promise for informing potential therapeutic approaches targeting age-related disorders of the human lens.

## Materials and methods

### Animals

Mice were housed and treated following the guidelines of the ARVO Statement for the use of animals in ophthalmic and vision research, and approved by the animal care and ethics committee at Zhongshan Hospital, Fudan University, Shanghai, China. Two age groups of mice, young (3-month-old) and aging (18-month-old) C57BL/6J male mice were supplied by Shanghai Bikai Keyi Biotechnology Co., Ltd. (Shanghai, China). All mice were housed at the animal facility under a 12/12 h light-dark cycle, and were given standard diet and water ad libitum.

Before administering different treatments, mice with abnormal body weight and appearance (e.g., eye closure) were excluded from the test. Preliminarily, all the mice were lightly anesthetized and their eyes were cyclopleged with 1% tropicamide ophthalmic solution. A-scan ultrasonography (KN1800; Kangning Medical Device Co., Ltd., Wuxi, China) was employed to screen axial ocular dimensions [8], thus excluding the cases of anisometropia. Lens examination was conducted directly with the naked eye to detect opacity and position, thereby excluding cases of cataracts. Additionally, abnormal mental states, such as mice displaying irregular swimming patterns or persistent floating, were also excluded.

For each group, RNA-seq and qPCR were conducted on the right eyes of six mice. Specifically, three out of the six were randomly selected for RNA-seq analysis, while the remaining three were used for qPCR validation. Additionally, two out of the six left eyes were randomly chosen for histological testing.

### Mouse exercise procedure

All the mice were divided into three groups (YS, AS and AE, *n* = 6 in each group). The visual representation of the sequential stages of the swimming procedure was depicted in the flowchart (Supplementary Fig. 1). In order to confirm the impact of physical activity on aging mice, the swimming exercise was carried out on the mice belonging to the AE group, guided by previous research [9, 10]. Briefly, swimming program was undertaken in a tank of water at 32 ± 1℃ with a water depth of 15 cm. Mice in AE group had one-week adaptive swimming (5 min a day, 3 times a week). The formal exercise regimen was divided into two stages, each lasting four.

weeks. Mice in AE group engaged in swimming activities for 10 min a day, three times a week during the first phase. In the subsequent stage, the swimming duration was increased to 15 min a day, three times a week. Referring to the previous study [11], the water container was equipped with a constant water current generated by a central water pump to prevent mice from ceasing swimming. Additionally, another experimenter observed nearby, ready to intervene if any mouse stopped swimming. Videos are available in the supplementary materials. All exercise sessions were performed during 9:00 AM to 10:00 AM. Mice were wiped with a dry towel and dried by a heater after swimming. To minimize potential confounding influences of stress or environmental enrichment, mice in the YS and AS groups were placed in an empty swimming tank alongside mice in the exercise group for a comparable period. After the 9-week of intervention, mice of YS group were sacrificed at age of 5 month, and mice of aging groups (AS and AE) were sacrificed at age of 20 months.

### RNA extraction and transcriptome sequencing

RNA was extracted from 1 µg of samples following the standard protocol of Shanghai Applied Protein Technology. The RNA quality was assessed using a Nanodrop ND-2000 system and an Agilent Bioanalyzer 4150 system. A mRNA-seq library prep kit was used to prepare the samples for transcriptome sequencing. mRNA was extracted from the total RNA using oligo magnetic beads. First and second strand cDNA were synthesized from the fragmented RNA using reverse transcriptase, DNA polymerase, and RNase H. The double stranded cDNA fragments were prepared for PCR amplification. A final cDNA library was obtained after PCR enrichment. The library was sequenced on an Illumina Novaseq 6000 or MGISEQ-T7 platform, generating 150 bp paired-end reads.

The sequencing data generated on the Illumina or BGI platform was used for bioinformatics analysis. Clean data was obtained by removing low quality reads, reads containing adapters, and poly-N reads using in-house scripts. Whole procedure was conducted through the FastQC tool (http://www.bioinformatics.babraham.ac.uk/projects/fastqc/).

### Expression and function analysis based on transcriptomics

RNA-seq data were aligned to the Ensembl Mus musculus reference transcriptome by HiSat2 (version 2.1.0). The clean reads were separately aligned to the reference genome using an orientation mode to map reads to exons, intergenic regions and introns. After mapping the reads to each gene, the read counts were obtained using FeatureCounts (http://subread.sourceforge.net/). Gene expression levels were quantified using FPKM based on gene length and number of reads mapped to each gene.

Gene set enrichment analyses (GSEA) were used to assess the Kyoto Encyclopedia of Genes and Genomes (KEGG) annotation enrichment. The result was imported into the javaGSEA app, and subsequently used to assess KEGG gene set expression between groups. Enrichment scores and p values were calculated using a default algorithm with 10,000 permutations. Gene set size filters were set at a minimum of 10 and a maximum of 500. Significantly enriched gene sets were defined as gene sets with a|normalized enrichment score (NES)| > 1, nominal p value < 0.05 and FDR < 0.05. Differential expression analysis was performed using DESeq2 (http://bioconductor.org/packages/release/bioc/html/DESeq2.html) to generate a volcano plot visualizing the amount and distribution of differentially expressed genes (DEGs). DEGs with an absolute log2 fold change (FC) over 1 and adjusted-p value below 0.05 were considered significantly differentially expressed [12]. Employing STRING database (Version: 12.0, https://www.string-db.org/), protein-protein interactions (PPI) analysis was conducted to demonstrate interactions between proteins translated from target genes. Minimum required interaction score = 0.4.

### Tissue histopathological and immunohistochemistry (IHC)

The mice were sacrificed and the lens were removed for histology verification. After immersed in formalin, dehydrated with ethanol gradient, the lenses were embedded in paraffin. Then samples were cut into 5 μm thickness and stained with hematoxylin and eosin (H&E) followed by observing under a brightfield microscope (Olympus, JP). H&E and IHC slides were taken from two mice in each group, and 3 slides were taken from each lens tissue. For H&E staining, three fields per slide were recorded to calculate the numbers of epithelial cells.

The paraffin sections were dewaxing to water utilizing xylene and anhydrous ethanol. Antigen retrieval was performed by placing the sections in EDTA antigen retrieval buffer (pH 8.0) and heating followed by natural cooling. Then, the sections were placed in 3% hydrogen peroxide solution to block endogenous peroxidase. 10% goat serum was added dropwise in the immunohistochemistry frame to uniformly cover the tissue, and incubated at room temperature for blocking. The sections were then incubated with a polyclonal antibody specific for CIART (abs100662, Absin, China). DAB chromogen solution was used for coloration. Finally, the cell nuclei were counterstained with Harris hematoxylin, followed by dehydration and mounting with neutral tree wax.

### RNA extraction, cDNA preparation, and quantitative RT-PCR

The lens RNA was extracted using a standard TRIzol-based method. Lenses were retrieved from mice using sterilized RNase-free instruments and stored at -80 °C immediately. Paired lenses from the same mouse were considered as one sample to go through the whole procedure. First, 1 ml of TRIzol was added to each sample and grinded at 70 Hz for 15 min (JXFSTPRP-CL, Jingxin Technology, SH). Then, 200 µL of chloroform was added, vortexed, and centrifuged at 12,000 g for 15 min at 4 °C. The aqueous phase (500 µL) was transferred to a clean tube and 600 µL of isopropanol was added. The mixed tube was put at -80 °C for 12–24 h in order to achieve complete RNA sedimentation. Thereafter, the sample was centrifuged at 12,000 g for 10 min at 4 °C followed by discarding the supernatant. The residue pellet was washed with 600 µL of precooled 75% ethanol, centrifuged at 7,500 g for 10 min at 4 °C and the supernatant was discarded. The pellet was dried for 20 min and resuspended in 10 µL of RNase-free water.

The extracted RNA was treated with a PrimeScript RT Master Mix reverse transcription reagent kit (RR036, TAKARA, JP) to synthesize cDNA. Quantitative RT-PCR was performed with specific primers for *Ciart*, *Crispld2*, *Igfl3*, and *Dpf3*. The *Actinβ* gene was used as an internal control because of its stability and ready expression. Supplementary Table 1 included the sequence of the primer used in qPCR analysis. For RT-qPCR, 2 µL of cDNA transcribed from lens RNA, 18 µL of a mix consisting of RNase-Free water, primer and 2X SYBR Green Pro Taq HS Premix (Rox Plus) (AG11718, Accurate Biology, CN) were used in a final reaction volume of 20 µL performed in a two-step Real-Time PCR System. Threshold cycle values and the ΔΔCt value of YS, AS and AE groups were analyzed in unpaired t-tests (YS vs. AS, AS vs. AE). The analysis was performed using GraphPad Prism 9 software.

### Statistical analysis

P values of the overlaps between two different sets of genes were calculated by using hypergeometric probability test (http://nemates.org/MA/progs/overlap_stats.html). IBM SPSS Statistics 20 (IBM SPSS, New York, USA) was used to analyze data, and GraphPad Prism 8 (GraphPad Software Inc., San Diego, CA, USA) was used to draw figures. Independent t-tests or Mann- Whitney U tests were utilized for normally or non-normally distributed data comparisons between two groups. Data were presented as mean ± Standard Deviation (SD). Differences were considered statistically significant when *p* < 0.05.

## Results

### The comparison of H&E staining in different lens group

**Fig. 1:**
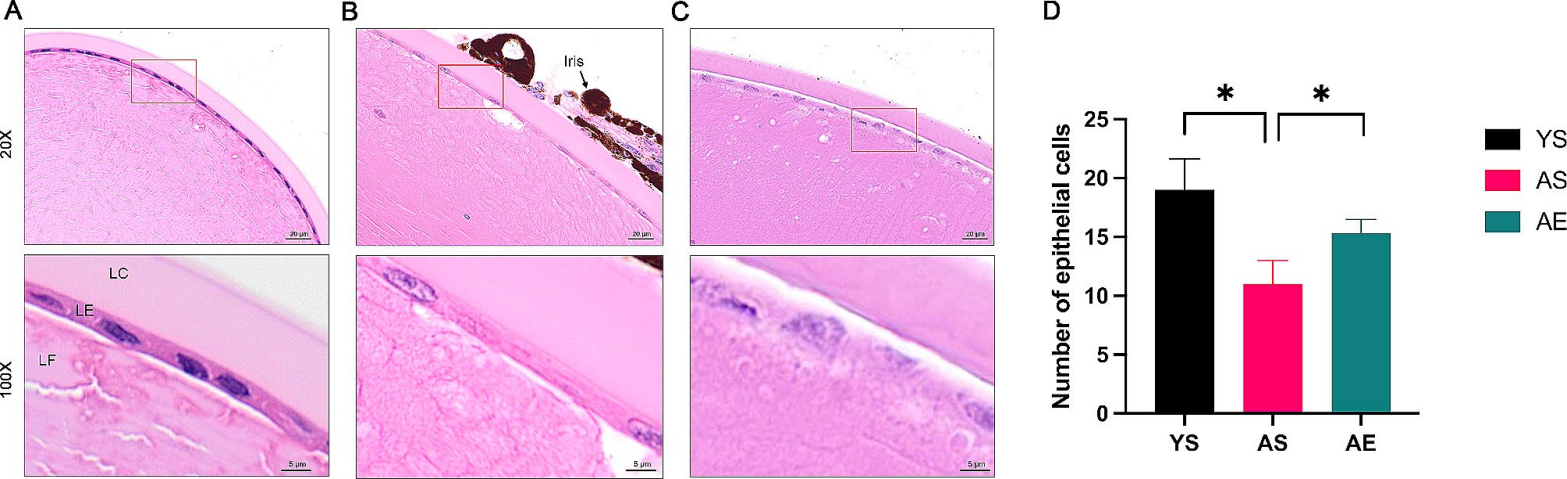
Histomorphological observation of the lens in the different groups. (**A**) Young sedentary group. (**B**) Aging sedentary group. (**C**) Aging exercise group. (**D**) Numbers of epithelial cell in different groups. Magnification = 40× and 100× as indicated. LC, lens capsule. LE, lens epithelium. LF, lens fibers. *p < 0.05

We firstly conducted H&E staining to observe the morphology of the lens (Fig. 1). In the YS group (Fig. 1A), the lens epithelial cells could be observed under complete phacocyst which disappeared posteriorly and receded to the equatorial circumference. The lens fiber layers were regularly arranged. Fibrocytic cells, derived from the epithelial precursors, were discerned progressing inward in a stepwise style, establishing the superficial cortical stratum. Relative to the YS group, the AS group (Fig. 1B) demonstrated a considerable decline in epithelial cell number. The cell allocation was relatively irregular and vacuolation appeared around the cells. Besides, the lens fiber layer was disorganized with irregular pore formation. Contrasted with AS group, a lightly-stained cytoplasm also could be seen in AE group and (Fig. 1C), but there was a noticeable reduction in pathological damage. The comparison between numbers of epithelial cells was demonstrated in Fig. 1D. Also, the decrease in the number of epithelial cells was mitigated and the lens fiber layers were more tightly packed.

### Overall changes of pathways induced by swimming exercise in aging lens unveiled by GSEA

**Fig. 2:**
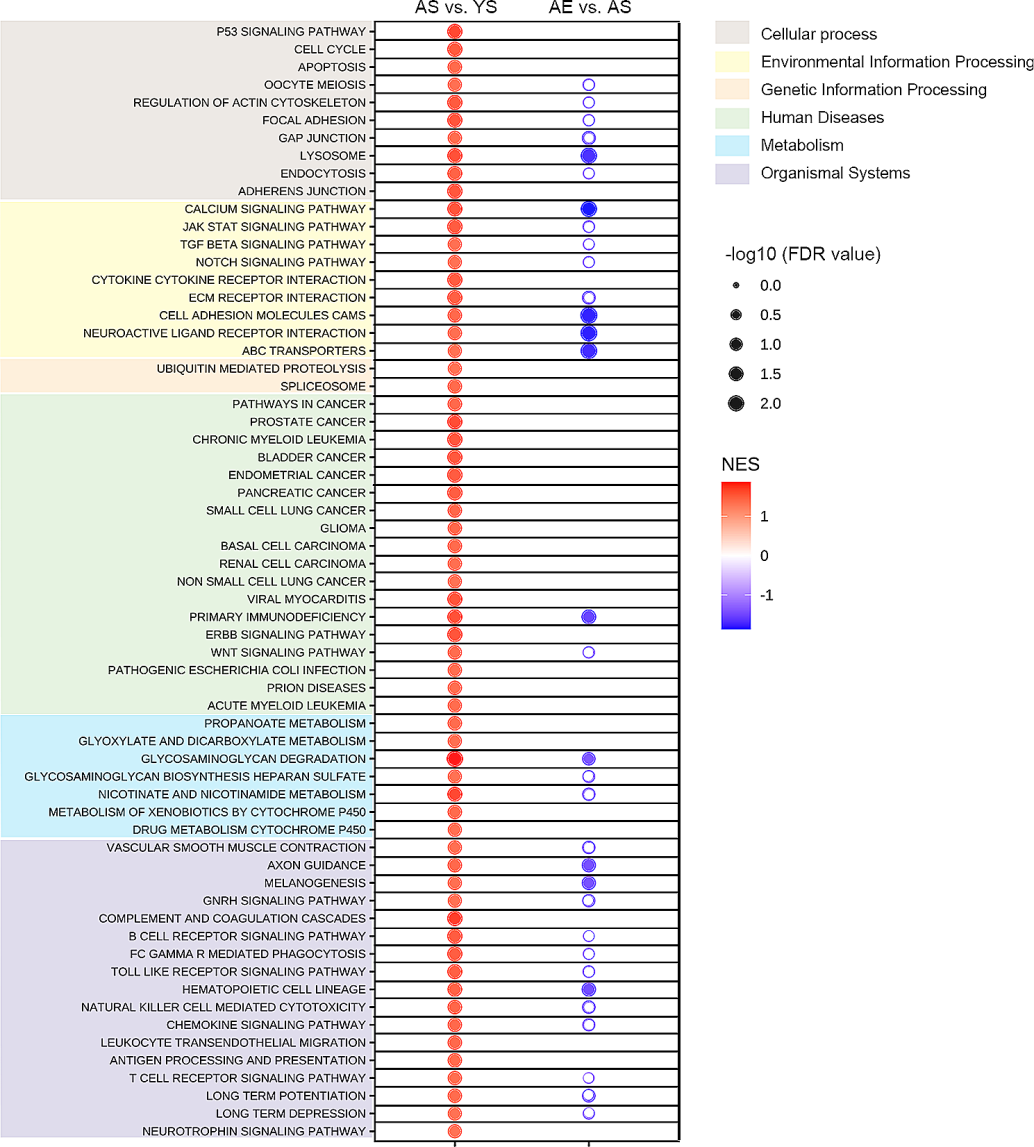
The bubble chart of KEGG pathway enrichment analysis by GSEA. The larger the bubbles were with smaller FDR value and greater significance. Red bubbles indicated the relatively upregulation in AS compared with YS group, and blue bubbles indicated the relatively downregulation in AE compared with AS group. *N* = 3/group

Firstly, we performed Principal Component Analysis (PCA) on the expression levels of all detected genes between AS and YS groups, which indicated an acceptable within-group differences and significant differences between groups (Supplementary Fig. 2). GSEA analysis did not require specifying thresholds (p value or FC) to filter DEGs. Performing GSEA analysis before DEG filtering maximized analytical efficiency and allowed us to compare AS/YS and AE/AS conditions [13, 14]. Inputting FPKM values and utilizing the GSEA procedure, we examined changes in KEGG pathways that occurred during the aging process, as well as the modifications that regular exercise brought to countering aging effect. Compared to the YS group, a total of 63 upregulated aberrant pathways were identified in the AS group (|NES| > 1, nominal p value < 0.05 and FDR < 0.05). Organized based on KEGG pathway classification, these 63 pathways could be classified into six prominent categories, encompassing cellular processes, environmental information processing, genetic information processing, human diseases, metabolism, and organismal systems. The NES and FDR values of the different pathways from various categories were visualized (Fig. 2, AS vs. YS). Detailed information was available in Supplementary Table 2. The most pronounced alterations due to aging (indicated by the deepest red color) were glycosaminoglycan degradation (NES = 1.87), complement and coagulation cascades (NES = 1.78) and p53 signaling pathway (NES = 1.73). Meanwhile, the categories of human diseases and organismal systems contained the most numerous assemblages of represented pathways. Within the realm of human diseases, the altered pathways were predominantly associated with cancer, encompassing ‘prostate cancer’, ‘chronic myeloid leukemia’ and ‘bladder cancer’. Meanwhile, under the organismal systems category, the prevalent changes were associated with modifications within the immune system, involving pathways such as ‘antigen processing and presentation’, and ‘B cell receptor signaling pathways’.

Applying the same approach, we conducted an assessment of the dysregulated KEGG pathways between AE group and AS group. As a result, swimming exercise significantly downregulated a total of 25 KEGG pathways (Supplementary Table 3). Among these, 10 pathways, approximately half of the total, coincided with the aberrant pathways that underwent alterations during the aging process (Fig. 2, AE vs. AS). In addition, swimming exercise led to a notable improvement in the majority of pathways within the categories of ‘cellular process’ and ‘environmental information processing’ that had been altered by the aging process. The three pathways with the most evident distinctions characterized by the deepest blue color (lowest NES) were: calcium signaling pathway (NES = -1.87), neuroactive ligand receptor interaction (NES = -1.85), cell adhesion molecules (NES = -1.84).

### Effects of swimming exercise on the DEGs of the aging lens

**Fig. 3:**
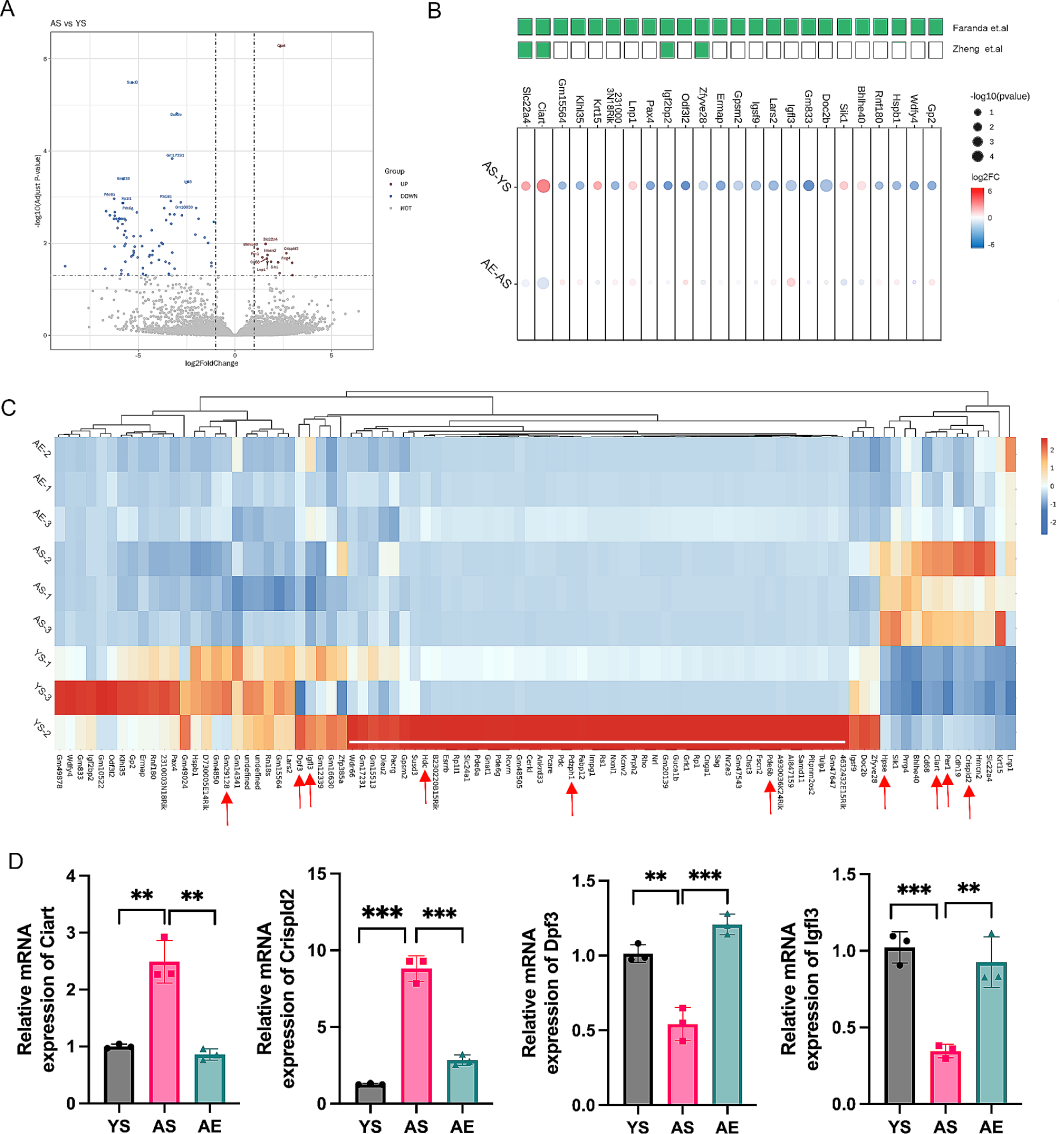
Effects of swimming exercise on the DEGs of the aging lens. (**A**) The Volcano plot of DEGs. The X axis represented transcript difference (log2-transformed fold changes), and the Y axis represented the corresponding -log10-transformed adj-p values. Red dots indicated significantly up-regulated genes, blue dots denoted significantly down-regulated genes, and gray dots symbolized proteins with no significant change. (**B**) The bubble map of the overlapping DEGs with previous articles. The green boxes in the first row represent the genes that overlap with Faranda et al., while those in the second row represent the genes that overlap with Zheng et al. The larger the bubbles were with smaller adjusted-p value and greater significance. Red bubbles indicated the relatively up-regulation in AS compared with YS group, and blue bubbles indicated the relatively down-regulation in AE compared with AS group. (**C**) The hierarchical cluster analysis contained values of the 92 DEGs in YS, AS and AE groups. The red arrows represented the genes that exhibited significant changes in both the AE (vs. AS) and AS groups (vs. YS). (**D**) Validation of the mRNA expression levels. Data represented mean ± SD. Significant differences between groups were noted by **p < 0.01, ***p < 0.001, ****p < 0.0001. *N* = 3/group

Meeting the established criteria of|log2(FC)| > 1 and an adjusted-p value < 0.05, the analysis of the transcriptome between the YS and AS groups revealed a total of 92 differentially expressed genes (DEGs). Among these DEGs, 13 were found to be upregulated, while 79 were downregulated (Fig. 3A). The detailed expression level of DEGs were in Supplementary Table 4. In order to assess the coherence of our data, we conducted a comparative analysis with prior transcriptomic studies [15, 16]. We employed a bubble plot to visualize the overlapping DEGs, considering both the adjusted-p value and FC values (Fig. 3B). In the study by Zheng et al. ^[16]^, they utilized chromatin-immunoprecipitation-based deep sequencing (ChIP-seq) to compare the lens of mice between those aged one month and 27 months. Their findings emphasized the significant role of histone H3 lysine 4 tri-methylation (H3K4me3) in the aging process, identifying a total of 613 promoter peaks that were markedly affected. In our research, 4 out of the 92 DEGs (*Zfyve28*, *Igf2bp2*, *Ciart*, and *Slc22a4*) were among the 613 promoter peaks in Zheng et al.’s study (p value = 0.19, representation factor = 1.8, hypergeometric test). Besides, the observed changes in direction of gene expression regulation all corresponded to the predictions by Zheng et al. Additionally, through RNA-seq analysis, a recent study conducted by Faranda et al. ^[15]^ unveiled significant alterations in lens epithelial cell and fiber cell genes between mice aged 3 months and those aged 24 months. Upon comparing the DEGs identified in Faranda et al.’s study with our own research, a shared pool of 28 genes was discovered (p value = 1.865e-20, representation factor = 9.8, hypergeometric test). Among these shared genes, *Zfyve28*, *Igf2bp2*, *Ciart*, and *Slc22a4* were also found to overlap with the findings from Zheng et al.’s work.

Having identified the 92 DEGs through comparison between the AS and YS group, we proceeded to compare the expression levels of these 92 DEGs in the AE group. The heatmap depicted relative expression level of each gene across the three groups (Fig. 3C). Out of the 92 genes experiencing changes due to the aging process, the swimming exercise was observed to notably mitigate the expression of 10 genes (AE vs. AS group,|log2(FC)| > 1 and adjusted-p value < 0.05), constituting roughly 10% of the total. In details, after the swimming exercise, the expression of 5 genes (*Hpse*, *Ciart*, *Crispld2*, *Per1* and *Gm29128*) were observed to be downregulated, while the other 5 genes (*Pdzph1*, *Igfl3*, *Pde6b*, *Hdc* and *Dpf3*) were found to be upregulated. Additionally, the clustering analysis of the heatmap indicated that genes primarily from Wdr66 to 4632432E15Rik (indicated by the white bar in the heatmap) exhibited abnormally high expression in YS-2, leading to excessive within-group variation. While statistical analysis can detect these differences, the discussion of these genes might be scaled back to enhance the feasibility of the data outcomes. To validate the results of transcriptome, a set of three genes was selected for qPCR analysis (Fig. 3D).

### *Ciart*: a key player in aging and its alleviation through swimming exercise

**Fig. 4:**
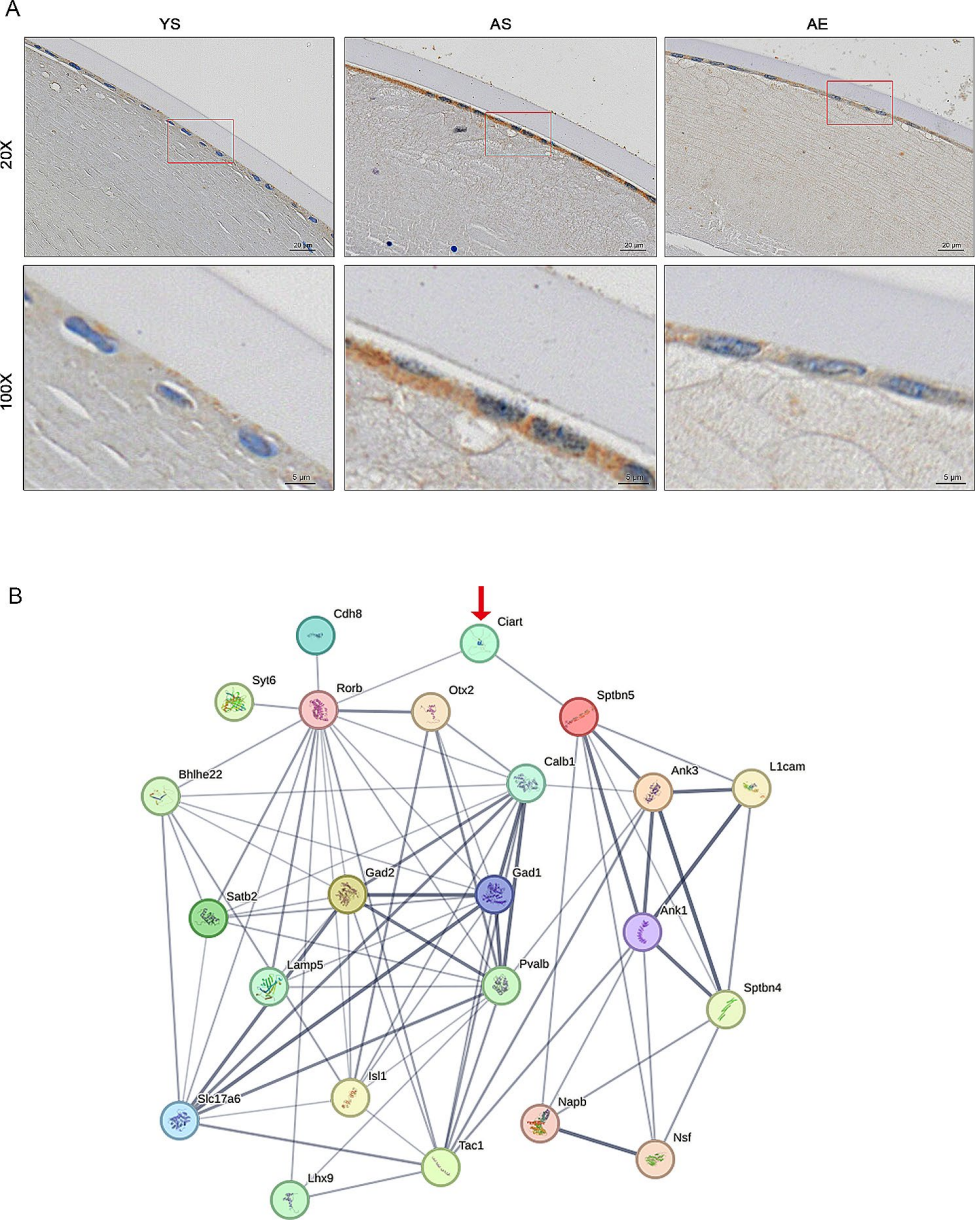
The role of ***Ciart*** in the aging process and swimming exercise. (**A**) The IHC staining of CIART in different groups. YS, Young sedentary group. AS, Aging sedentary group. AE, Aging exercise group. Magnification = 40× and 100× as indicated. (**B**) Constructed protein-protein interaction (PPI) network. Minimum required interaction score = 0.4. Line thickness indicated the strength of data support by STRING database. *N* = 3/group

Based on the aforementioned findings, *Ciart/*CIART (Circadian associated repressor of transcription) was acknowledged in the aging process, and it could be also notably influenced by swimming exercise. Hence, we proceeded to conduct IHC staining to validate its distinct protein expression levels and investigate the distribution within the lens. (Fig. 4A). As a result, in the YS group, we observed its focal positive expression in the lens epithelium, minimal expression in lens fibers, and no expression in the lens capsule. Compared to the YS group, the expression of CIART protein in the AS group was significantly up-regulated, with the majority of the expression observed in the lens epithelium. The IHC staining of the lens epithelium in the AE group revealed a dramatically decreased expression of CIART protein in comparison to the AS group. The results of IHC abundance were consistent with mRNA expression results. Additionally, to illustrate the interactions of *Ciart* with other genes that exhibited significant alterations between the AS and AE groups, we constructed a PPI network in Fig. 4B. The DEGs between the AS and AE groups from this analysis was in Supplementary Table 4, with genes involved in PPI network highlighted. In this section, PPI (with 22 nodes and 73 edges) were constructed by first identifying two genes directly associated with Ciart: Rorb and Sptbn5. Subsequently, additional genes associated with Rorb and Sptbn5 were identified, resulting in a total of 23 genes used for PPI construction. In the STRING database, *Ciart* had a direct connection with *Rorb* (Retinoid-related orphan receptor B) and *Sptbn5* (Spectrin Beta), while *Rorb* and *Sptbn5* interacted with a larger number of DEGs. Among the 92 DEGs in our study and the 23 of DEGs in Ciart protein interactions network, only Ciart was overlapped, resulting in *p* = 0.08 (with Representation factor = 11.8, hypergeometric test).

## Discussion

While the advantages of physical activity for the well-being of elderly individuals had been widely recognized, the mechanisms underlying how exercise ameliorates pathological alterations in the aging lens remained to be fully elucidated. The results of the present study demonstrated the successful suppression of age-induced changes via swimming exercise. Utilizing RNA-seq, we could identify specific molecules and pathways that could have played pivotal roles in facilitating this restorative process. To the best of our knowledge, these data provided the first direct evidence indicating the potential of exercise modifying the effects of aging on the mouse lens transcriptome. The mice in the YS group were aged between 3 and 5 months, a phase selected due to the attainment of sexual maturity and completion of eye development as mature adults. The aged mice in both the AS and AE groups were between 18 and 20 months old, which corresponded to a stage equivalent to human in their late 60s [17]. Compared with formal studies [9, 18], the intensity of the swimming exercise was kept at a relatively low level. This deliberate choice aligned with the clinical approach of safely prescribing exercise at a lower intensity level, particularly suitable for the aging population [19].

The lens was composed of ectodermal cells at different stages of differentiation. The most superficial epithelial cells were metabolically active while the deeper fiber cells that make up the most of lens (formed the nuclei lentis) were organelle-free as they continued to differentiate [1]. Compared to the YS group, the AS group exhibited disorganized epithelial cells and fibers, align with the documented age-related changes in murine lenses [20]. In contrast to the AS group, lens in AE group exhibited an improvement in both disorganized fibers and epithelial cell quantity, indicating that the swimming exercise could counteract the histopathological signs of aging lens. The epithelial cells were known as the principal cells of the lens throughout its life cycle. The presence of deformed epithelial cells in the AE lens suggests that the epithelial cells continued to proliferate and underwent intricate intracellular metabolic processes [21, 22], playing a role in repair and regeneration after exercise stimuli.

By utilizing the bioinformatics tool GSEA, we analyzed overall data trends, enabling us to investigate the directional changes of distinct pathways under conditions of exercise and sedentary. In comparison between the lenses of aging and young mice, we observed specific pathways that were known to play a crucial role in the development of age-related changing of the lens. For instance, p53 signaling pathway and apoptosis were major molecular events observed in human cataractous lenses [23]. TGF beta signaling pathways and extracellular matrix receptor interaction were known to lead to the occurrence of posterior subcapsular congenital cataract [24]. Furthermore, it could be seen that the directions of abnormal pathway changes in aging lenses were consistently up-regulated, while exercise predominantly down-regulated these effects. Upon comparing these groups, swimming exercise had the potential to specifically target certain types of abnormal processes associated with aging. The three most prominently altered pathways were ‘calcium signaling pathway’, ‘cell adhesion molecules’ and ‘neuroactive ligand receptor interaction’, all pertained to ‘environmental information processing’ category. Suggested by previous studies, the calcium homeostasis and signaling were key disciplines in lens research [25]. During aging, the accumulation of endoplasmic reticulum (ER) stress activated calcium-dependent proteases, leading to the fragmentation of different enzymes and proteins and the impairment of normal lens function [26]. Exercise had been shown to maintain the hemostasis of calcium in other tissues [27, 28], and our research findings further confirmed its role within the aging lens. Furthermore, the pathway of ‘cell adhesion molecules’ was previously found to regulate human cataract [29, 30] and its responsiveness to exercise had been noted in other diverse clinical contexts besides the eye [31]. Surprisingly, the neuro-related pathways were also significantly involved, including ‘neuroactive ligand receptor interaction’ and ‘long term depression’. A previous article proposed that lens fiber cells shared regulatory characteristics similar to neuronal cells [32], but the exploration of the detailed role of neuro-related pathways within the lens was limited.

Through a comparative analysis of the lens transcriptome between young and aging mice, we uncovered 92 genes that significantly altered by age, with some of them being linked to the onset of cataracts. For instance, cataract onset has been observed in patients with *Nr2e3* [33, 34], *Rp1* [35] and *Hspb1* mutations [36, 37]. And *Pde6b* [38] and *Nrl* [39] were respectively identified as potential modifiers in *Fox3e3* (-/-) and *S100a4* (-/-) mice, expediting the progression of cataract development. Prior studies had also systematically investigated the aging mouse lens, enabling us to conduct a comparative analysis to identify shared characteristics. Despite discrepancy in mice gender and age between our study and a previous investigation ^4^, we identified a consist set of 28 genes displaying alterations. Moreover, using CHIP-seq analysis, another article identified 613 significantly changed promoter peaks by comparing H3K4me3 peaks between young and old lenses. Relative to the aforementioned study, we further observed an overlap of 4 genes within our result (*Zfyve28*, *Igf2bp2*, *Ciart*, and *Slc22a4*). This also demonstrated the age-dependent changes in H3K4me levels, emphasizing the need for deeper investigation into the role of these genes in the aging process. Furthermore, after a session of swimming exercise, we noted the subsequent modifications in these DEGs that had been experienced alterations during the aging process. Remarkably, the transcriptomic results demonstrated that exercise has the potential to alleviate around 10% of the aberrant gene expression induced by the aging process and Ciart was particularly noteworthy as it continued to play a significant role in this process.

Intriguingly, *Ciart* had been identified as a crucial transcription factor that played a significant role in governing the mammalian circadian clock [40, 41]. The upregulation of *Ciart*-mediated circadian rhythms has been found to be associated with neural system abnormalities in depression [42] and immune regulation abnormalities related to viral infections [43]. In our study, *Ciart* was significantly up-regulated in aging process, and this alteration was alleviated by swimming exercise. Previously, Lim et al. [44] proposed the concept that age-related disruptions in circadian rhythms might lead to disturbances in glutathione homeostasis and redox regulation, potentially resulting in the development of age-related lens cataracts. Our study further identified *Ciart* as the core circadian clock components. Expanding upon the earlier findings of GSEA analysis in our study, we assumed that the swimming exercise influenced *Ciart*-related circadian rhythms, which contributed to processing environmental information, including pathways such as ‘calcium signaling’, ‘cell adhesion molecules’ and ‘neuroactive ligand receptor interaction’. Eventually, we observed the exercise regulate aging-related morphological and transcriptomic changes. There is consensus that oxidative stress is the major contributing factor to age-related nuclear cataract formation, but oral administration of nutrients and antioxidants to date has proven to be largely ineffective [45]. Our research suggested that in addition to antioxidation, there were other aging and anti-aging mechanisms worthy of consideration as strategies for cataract drug development. Based on the growing number of literatures recognizing the relationship between circadian rhythm disturbances and aging [44, 46]. Our study could explore the current data in this field and guide to design new therapies based on this regulation.

By the analysis of PPI, this regulation was partially achieved through the control of *Rorb* and *Sptbn5*, two genes could be also identified from the multi-organoid analysis. *Rorb* was a member of nuclear hormone receptors that had some natural ligands including all-trans retinoic acid and other retinoids [47]. *Sptbn5* was regarded as a component of a widespread complex system that connects membrane proteins, lipids, and cytosolic factors with the essential cytoskeletal elements of the cell [48]. The roles of *Ciart*, *Rorb* and *Sptbn5* were previously indicated in various neurological disorders, including seizures, Parkinson’s disease and depression [42, 48, 49]. Expanding upon the earlier findings in our study, the investigation of *Ciart*-related circadian rhythms was warranted, and this connection held promise as a compelling direction for future research. Finally, it was important to acknowledge the limitations of our study. The sample size was relatively small, and we recognize the need for further validation through increased sample size in future studies.

## Conclusion

In summary, we investigated the changes occurring throughout the exercise and sedentary of aging mice at the morphological, omics and molecular levels. Our research integrated RNA-seq in YS, AS and AE groups, for a deeper understanding of age-related lens disorders and future strategies of molecular-level mitigation. We observed that *Ciart*-related pathways might have a protective effect on the aging process, which could be associated with the circadian rhythms.

### Electronic supplementary material

Below is the link to the electronic supplementary material.

Supplementary Material 1

Supplementary Material 2

Supplementary Material 3

Supplementary Material 4

Supplementary Material 5

Supplementary Material 6

Supplementary Material 7

## Data Availability

The transcriptome sequencing reads have been deposited in the NCBI sequence read archive (SRA) database with links to BioProject accession number PRJNA1072988. The datasets used and/or analysed during the current study available from the corresponding author on reasonable request.
