# Supplementary figures and images for: Swimming exercise reverses transcriptomic changes in aging mouse lens
Source: BMC Med Genomics. 2024 Mar 4;17:67. doi: 10.1186/s12920-024-01839-1 (PMC10913554; doi:10.1186/s12920-024-01839-1)

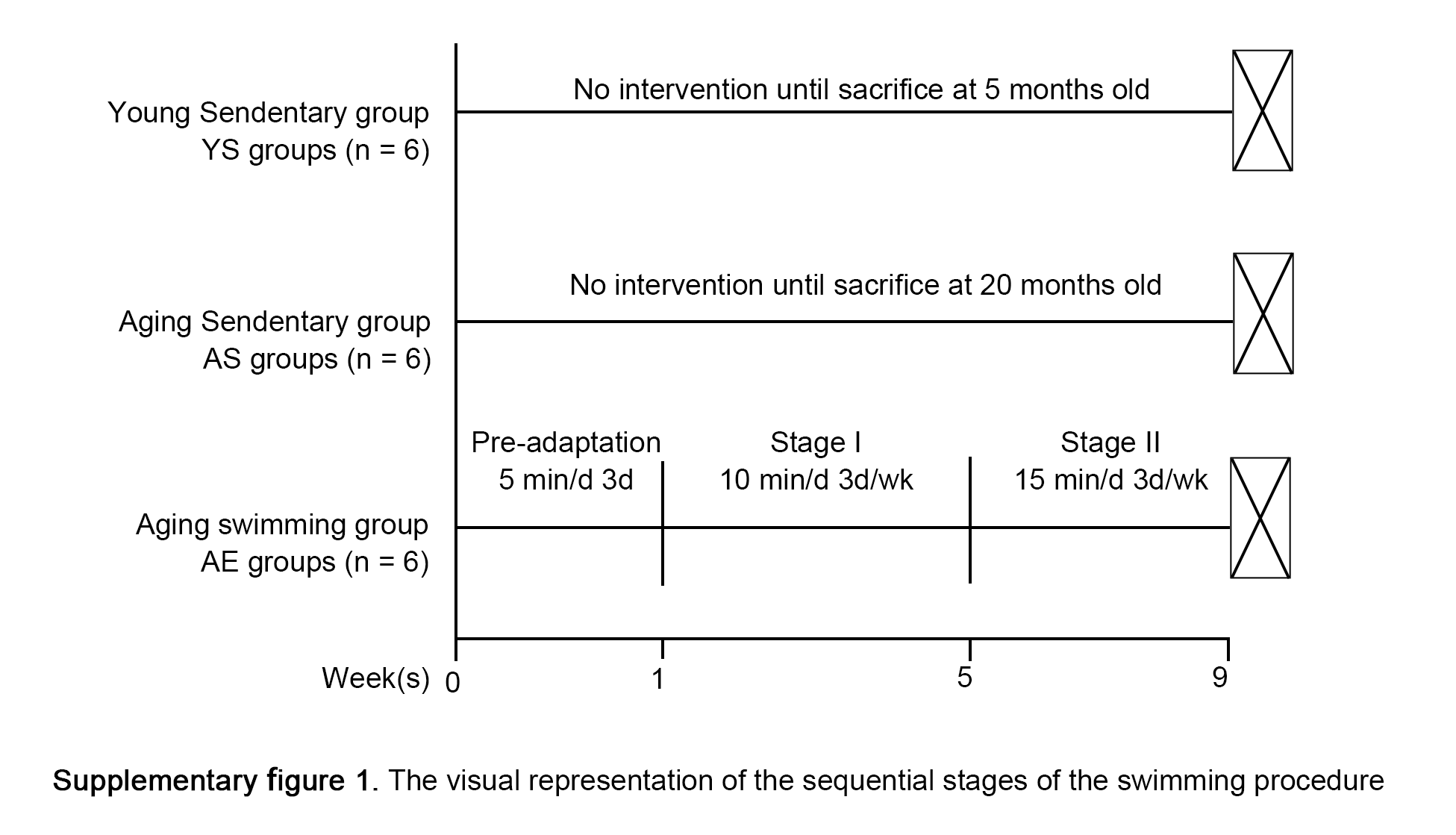

Supplement: Supplementary file 4 — Supplementary Material 4 [file 12920_2024_1839_MOESM4_ESM.tif]

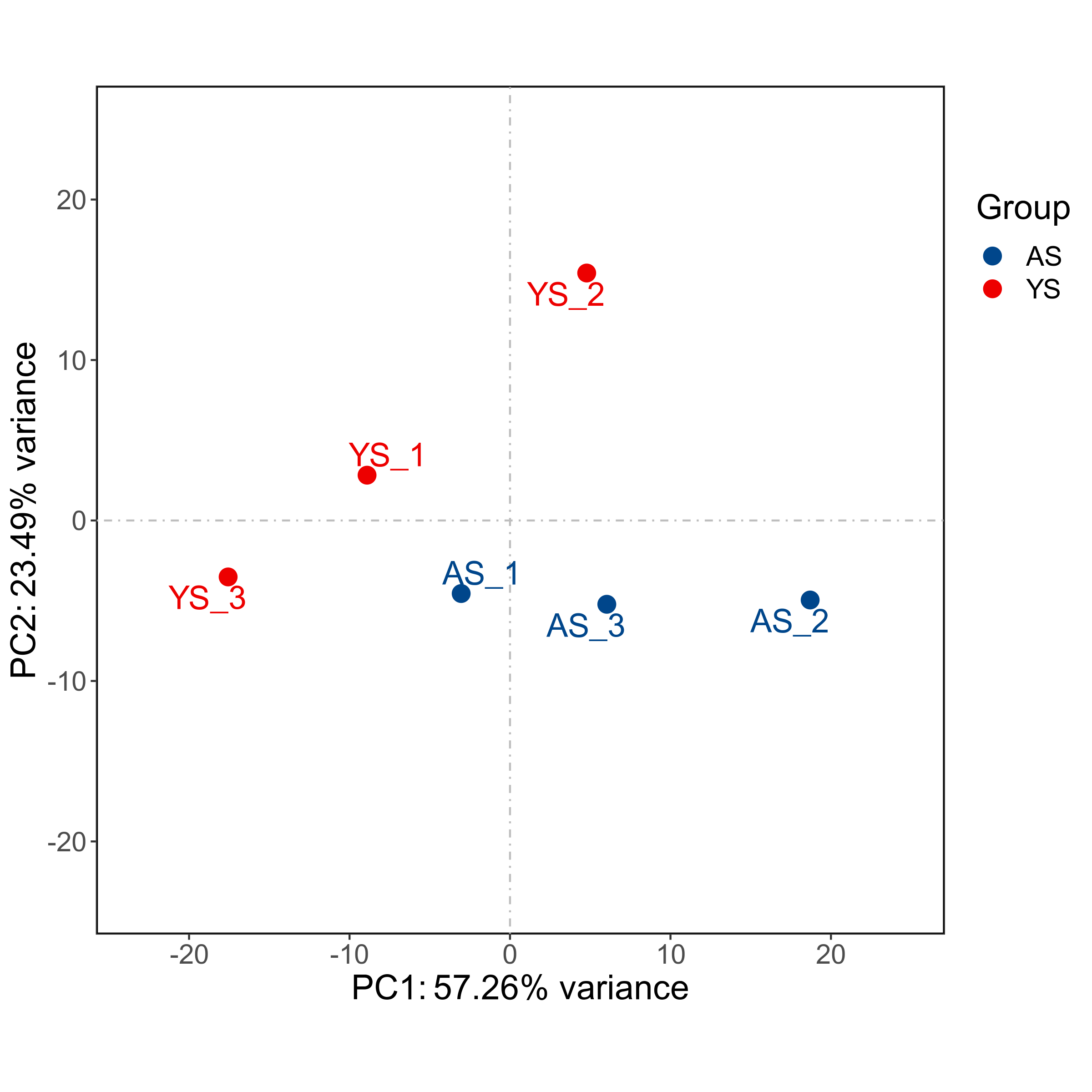

Supplement: Supplementary file 5 — Supplementary Material 5 [file 12920_2024_1839_MOESM5_ESM.png]

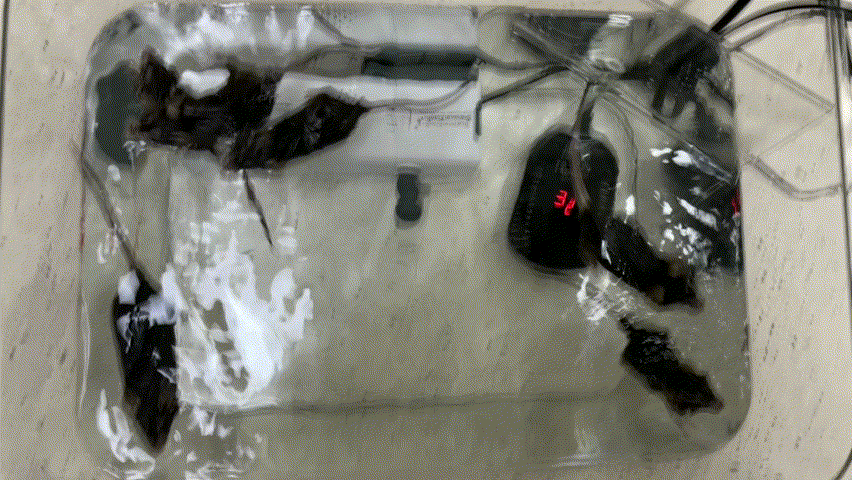

Supplement: Supplementary file 6 — Supplementary Material 6 [file 12920_2024_1839_MOESM6_ESM.gif]
